# Established and Emerging Roles of Epigenetic Regulation in Diabetic Cardiomyopathy

**DOI:** 10.1002/dmrr.70081

**Published:** 2025-08-19

**Authors:** Adam Russell‐Hallinan, Narainrit Karuna, Frank Lezoualc'h, Giuseppe Matullo, Hana Baker, Monique Bernard, Yvan Devaux, Lina Badimon, Gemma Vilahur, Jennifer Rieusset, Geneviève A. Derumeaux, Chris J. Watson

**Affiliations:** ^1^ Wellcome‐Wolfson Institute for Experimental Medicine Queen's University Belfast Belfast UK; ^2^ Institute of Metabolic and Cardiovascular Disease INSERM University Toulouse III‐Paul Sabatier Toulouse France; ^3^ Department of Medical Sciences University of Turin Turin Italy; ^4^ Diabetes and Complications Research Lilly Research Laboratories Eli Lilly and Company Indianapolis Indiana USA; ^5^ Aix‐Marseille University CNRS CRMBM Faculté de Médecine Marseille France; ^6^ Cardiovascular Research Unit Department of Precision Health Luxembourg Institute of Health Strassen Luxembourg; ^7^ Cardiovascular Program‐ICCC IR‐Hospital de la Santa Creu I Sant Pau Barcelona Spain; ^8^ Laboratoire CarMeN UMR INSERM U1060/INRA U1397 Université Claude Bernard Lyon1 Lyon France; ^9^ Department of Physiology INSERM U955 Université Paris Est Créteil (UPEC) AP‐HP Henri Mondor Hospital FHU SENEC Faculté de Santé de Créteil Créteil cedex France

**Keywords:** BET proteins, circRNAs, diabetic cardiomyopathy, DNA methylation, epigenetics, epitranscriptomics, histone modifications, lncRNA, miRNA

## Abstract

An increasing number of individuals are at high risk of type 2 diabetes (T2DM) and its cardiovascular (CV) complications, which challenges healthcare systems with an increased risk of developing CV diseases. Patients with T2DM exhibit a unique cardiac phenotype termed diabetic cardiomyopathy (DCM). DCM usually involves complex and multifactorial pathogenic drivers, including myocardial inflammation, fibrosis, hypertrophy, and early diastolic dysfunction, which potentially evolve into systolic dysfunction and heart failure. There is a lack of effective treatments for DCM on the basis of the complexity of the disease per se and poor understanding of the mechanisms behind disease development and progression. Despite the considerable research attention on the onset of DCM development and progression, understanding of the full spectrum of pathogenic mechanisms has not yet been fully deciphered. Epigenetic alterations, including DNA methylation, histone modifications, bromodomain extra‐terminal (BET)‐containing reader proteins, and RNA‐based mechanisms (e.g., miRs, lncRNAs, circRNA), are significantly associated with the initiation and evolution of DCM, particularly in the early stage. In this review, we provide insights into the evidence of epigenetic alterations related to DCM development and progression characteristics. Furthermore, the uniqueness of epigenetic changes in DCM in specific cell types within diabetic hearts is discussed. We also review epigenetic cooperation in the context of DCM development and epigenetic biomarkers related to DCM progression. With recent advancements in technology, epitranscriptomics‐related to DCM has been uniquely discussed. Finally, this review may provide new avenues for potential implications for future research and the discovery of novel treatment targets for preventing the onset and progression of DCM.

## 1 Diabetic Cardiomyopathy and Epigenetics

The prevalence of type 2 diabetes mellitus (T2DM) is increasing worldwide, and this diabetes epidemic is afflicting all ages, genders, and socioeconomic classes, leading to frailty and compromising healthy trajectories [1]. Epidemic increase in T2DM prevalence is linked to major changes in lifestyle and dietary habits, including high‐calorie and high‐fat diet (HFD) and sedentary attitudes [2]. These environmental triggers are significantly modulated by genetic predisposing factors, orchestrating epigenomic pathways that regulate gene expression and may contribute to cell and organ function modifications. For example, the control of inflammation by reprogramming immune cells has already been shown, demonstrating how epigenetic sensing leads to changes in chromatin accessibility and histone tail modification (methylation and acetylation) that favour inflammatory gene expression [3]. Thus, epigenomic mechanisms are at the interface between metabolic disorders and inflammation [4] and are implicated in inflammatory processes throughout T2DM development, which occurs when insulin secretion from pancreatic beta cells cannot sufficiently be increased to compensate for insulin resistance. Indeed, the development of T2DM is a slow process, preceded by a long and silent prediabetes phase with subtle organ complications that often remain undiagnosed and difficult to prevent at the medical level. For instance, recent data from the CARDIA study have provided good evidence for the diagnosis of diabetic cardiomyopathy (DCM), demonstrating that subtle systolic and/or diastolic myocardial abnormalities occur early in DCM development and precede the onset of overt cardiomyopathy [5]. These observations suggest that identifiable triggering factors are involved in the deterioration process, which could be subjected to interceptive actions. Notably, cardiovascular (CV) complications are the main causes of morbidity and mortality in T2DM patients, accounting for about two‐thirds of overall deaths, as evidenced by the Framingham Heart Study [6]. One of the major CV complications in T2DM is the onset of DCM, originally described as an early diastolic dysfunction leading progressively to systolic dysfunction and heart failure without hypertension, coronary artery disease and valvular or congenital heart disease [7].

**FIGURE 1:**
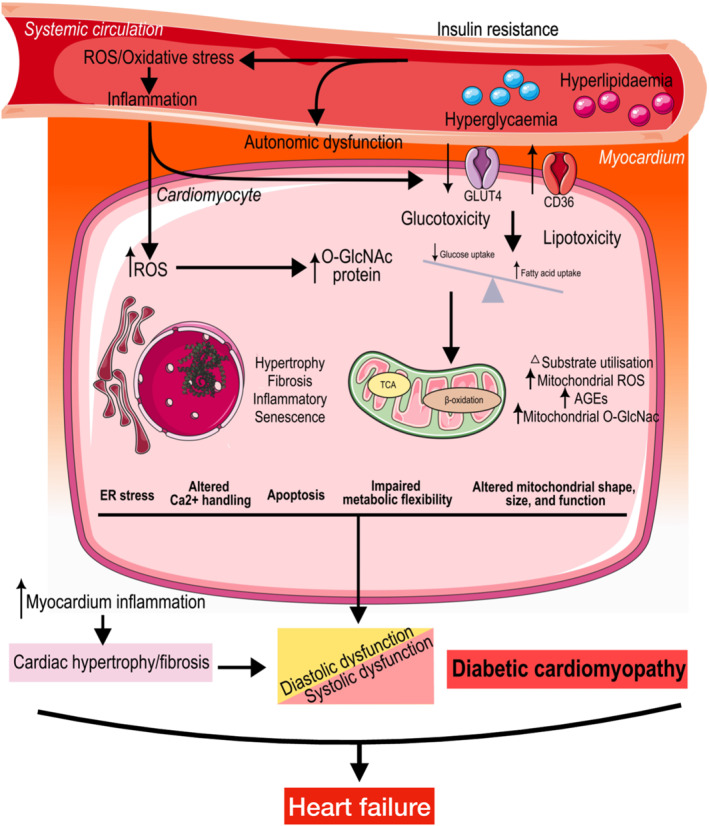
Contributing mechanisms related to cardiomyocytes that induce the pathobiology of diabetic cardiomyopathy. Type 2 diabetes mellitus with insulin resistance mediates systemic changes, including hyperglycaemia, hyperlipidaemia, and metabolic alterations. Systemic changes also interact with different signalling molecules, such as NADPH oxidase, to trigger the production of reactive oxygen species (ROS) or reactive nitrogen species. Dysfunctional mitochondria produce excess ROS, which increases oxidative stress. Oxidative stress is defined as an imbalance between inappropriate (e.g., excessive) ROS generation and the capacity for them to be degraded. Disrupted cell metabolism and oxidative stress can lead to endoplasmic reticulum (ER) stress, resulting in cardiomyocyte apoptosis and hypertrophy. Oxidative stress, ER stress, and inflammation can mutually activate and reinforce each other, driving these pathological processes. Furthermore, alterations in mitochondrial Ca^2+^ signalling cause abnormalities in cardiomyocyte Ca^2+^ handling and contractility. Impaired metabolic pathways, including abnormalities in substrate utilisation, mitochondrial function, AGE formation, and O‐GlcNAcylation, as well as changes at the level of insulin signalling, gene regulation, ER stress, autonomic dysfunction, and cardiac cell apoptosis, have been accepted as mediators of diabetes–induced cardiac remodelling and dysfunction. These contributing mechanisms are complex and multifactorial in the context of diabetes–induced cardiomyopathy. Collectively, these alterations contribute to cardiac impairments‐structural and functional changes, termed diabetic cardiomyopathy, potentially developing into HF over time. AGEs = Advanced glycation end products; CD36 = Cluster differentiation 36; ER = Endoplasmic reticulum; GLUT4 = Glucose transporter type 4; O‐GlcNAc = Oxygen‐linked β‐N‐acetylglucosamine; ROS = Reactive oxygen species.

DCM is now recognised as a distinct cardiac phenotype leading to ventricular remodelling and abnormal myocardial contractility, which correlate with multifactorial and complex molecular and cellular changes (Figure 1) [5, 8, 9]. Both insulin resistance and chronic hyperglycaemia contribute to impaired cardiac contractility and structure via reduced Ca^2+^ influx through L‐type Ca^2+^ channels, impaired PI3K/Akt pathway, accumulated reactive oxygen species (ROS), increased advanced glycation end products (AGEs) formation, increased lipotoxicity, and multiple potential mechanisms such as autophagy, chronic inflammation, and epigenetic mechanisms [10]. Importantly, epigenetics associated with chronic inflammation and deregulation of the immune system play a major role in the dysregulation of glucose homeostasis, which in turn promotes gene‐activating epigenetic changes and signalling events critical in the development and progression of CV complications [11, 12].

The role of epigenetic modifications in DCM is currently not well deciphered, but growing evidence supports that T2DM drives an epigenetic reprogramming of cardiac metabolism, impairing both metabolic flexibility and metabolic memory [13], contributing to the development of heart failure [11]. Thus, understanding the impact of epigenetic processes in the context of DCM will provide new insights into this complex disease and guide new therapeutic strategies for preventing or reversing DCM. Indeed, as recently reviewed [14], ‘epidrugs’ may represent a viable therapeutic approach for preventing diabetic‐related heart failure, given the widespread epigenetic modifications that have been uncovered [15]. However, the numerous epigenetic regulatory proteins and chromatin modifications have yet to be fully assessed for their roles in the control of cardiac fibrosis and remodelling process in DCM. Furthermore, the utility of integrating cross‐species epigenomics needs to be addressed to demonstrate the potential relevance of this approach for human studies on DCM [16]. Taken together, understanding and investigating epigenetic factors involved in DCM development may provide promising new avenues to address DCM and slow disease progression. In the following sections, we discuss epigenetic factors related to DCM and summarise evidence of epigenetic alterations targeting pathogenic drivers of DCM.

## 2 Epigenetic Mechanisms and Their Alterations in Diabetic Cardiomyopathy

Systemic metabolic dysfunction in DM hyperglycaemia, dyslipidaemia and associated glucotoxicity, lipotoxicity and oxidative stress are the major pathological mechanisms driving the development of DCM [17, 18]. These metabolic alterations are well established to predispose and influence pathological responses of resident and infiltrating myocardial immune cells that promote aberrant structural and functional cardiac changes in preclinical and clinical DCM [19, 20, 21, 22]. Underlying this pathological cellular behaviour involves dynamic changes at the molecular level of various gene regulatory mechanisms that enable cells to respond or adapt to metabolic insults [23]. Consequently, epidemiological and clinical studies have suggested that these alterations that occur as a result of cumulative metabolic stress are retained in the form of ‘metabolic or glycaemic’ memory, which can contribute to long‐term diabetic complications [11, 24, 25, 26]. Due to the clear association between environment, diet and transcriptional activity level on diabetes‐related complications, the influence of various epigenetic mechanisms and their contributions to dysfunctional gene regulation and subsequent aberrant cellular function in DCM is becoming increasingly apparent [27, 28, 29].

**FIGURE 2:**
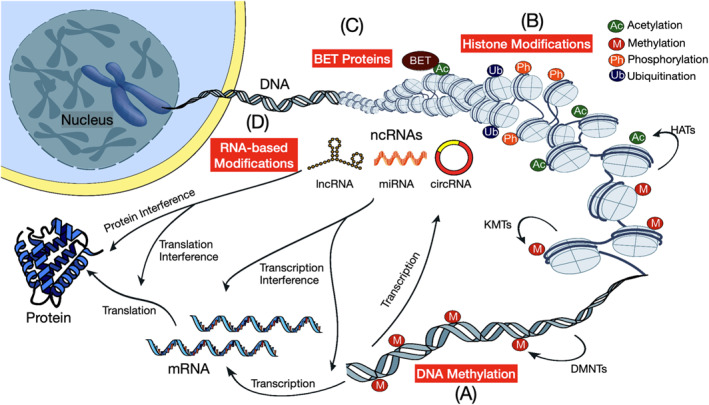
Landscape of location of epigenetic modifications. Epigenetics refers to the genetic influences that lead to altered gene expression without altering the DNA sequence. These epigenetic changes include DNA methylation, histone modifications, bromodomain and extra‐terminal domain (BET) proteins, or RNA‐based modifications (e.g., lncRNA, miR, and circRNA). (A) DNA methylation occurs predominantly at cytosine‐phosphate‐guanine (CpG) islands. This involves the addition of methyl groups to cytosines that are followed by guanine in the DNA sequence and is catalysed by DNA methyltransferases (DNMTs). DNA methylation regulates gene expression by recruiting proteins involved in gene repression or by inhibiting the binding of transcription factor(s) to DNA. (B) Histone complexes can undergo modifications at various positions on their tails, collectively forming what is known as the histone code. Histone tail acetylation by histone acetyltransferases (HATs) results in an open and accessible chromatin structure, facilitating transcription. Histone tail methylation, mediated by lysine methyltransferases (KMTs), can either activate or repress transcription. (C) Expression and activity of BET proteins are regulated at different levels, including transcription by 5‐hydroxymethylcytosine (5‐hmC) at the BRD4 promoter, messenger RNA (mRNA) stability by binding of microRNA (miRNA) to the 3′ untranslated region (UTR) of BRD4 mRNA transcripts, and translation by N^6^‐methyladenosine (m^6^A) modification of mRNA. (D) RNA‐based modifications (non‐coding RNAs; ncRNAs), including microRNAs (miRs), long ncRNAs (lncRNAs), and circular RNAs (circRNAs). These ncRNAs can govern epigenetic changes through several complex and multifactorial mechanisms as well as their sequences. miRs regulate gene expression by binding to complementary sequences on target mRNA, thereby inhibiting their translation. Expression of lncRNAs influences gene expression through diverse mechanisms, including chromatin remodelling, transcriptional co‐activation or repression, posttranscriptional modifications, and protein inhibition. circRNAs can function as miR sponges or decoys to regulate target mRNA. All these epigenetic regulations can interact with each other, indicating an intricate network to determine gene and protein expression. BET = Bromodomain and extra‐terminal domain; BRD4 = Bromodomain‐containing protein 4; DNMTs = DNA methyltransferases; HATs = Histone acetyltransferases; KMTs = Lysine methyltransferases; ncRNAs = Non‐coding RNAs.

Epigenetics is defined as heritable modifications occurring in the genome resulting in a change in gene expression without affecting the DNA sequence [30]. Epigenetic mechanisms include histone modifications (e.g., acetylation, methylation), bromodomain extra‐terminal (BET)‐containing reader proteins, DNA methylation and non‐coding RNA (ncRNA)‐based mechanisms (Figure 2). The latter encompasses small single‐strand ncRNA molecules named microRNAs (miRs), long non‐coding RNAs (lncRNAs), and more recently circular RNAs (circRNAs), which regulate their target genes at a post‐transcriptional level [31]. Of note, various components of the epigenetic machinery, such as the nicotinamide adenosine dinucleotide (NAD)‐dependent deacetylase, Sirtuin 1 (SIRT1), require intermediates of cellular metabolism for their enzymatic activity. In addition, changes in intracellular metabolism observed in T2DM can alter the expression of specific histone transferases, conferring widespread variations in epigenetic modification patterns [31, 32]. Research in the field of ncRNA has highlighted new mechanistic links between diabetes and DCM, with evidence proving the involvement of miRs and lncRNAs in the pathogenesis of DCM [33]. Adding complexity to the matter, specific epigenetic signals affecting the three main layers of epigenetic modifications convey to alter metabolic homeostasis and cardiac function in the setting of experimental and human diabetes [34]. Therefore, the identification of these complex epigenetic alterations might lead to the development of novel therapeutics targeting pathogenic mechanisms underlying the development of DCM.

## 3 DNA Methylation

DNA methylation is the enzymatic addition of a methyl group to the carbon 5′ position of a DNA cytosine ring, resulting in the formation of 5‐methylcytosine (5MeC) mediated by DNA methyltransferases (DNMT1, DNMT3A and DNMT3B) [29]. Regulation of gene expression by DNA methylation is primarily associated with transcriptional repression, occurring in the promoter region either directly or indirectly. DNA methylation can directly interfere with transcription factor binding to recognition sites in the promoter region or through the recruitment of co‐repressor proteins (e.g., methyl CpG‐binding protein 2 [MeCP2]) that promote the formation of a repressed chromatin state [35]. Outside of the promoter region, DNA methylation at intergenic regions and gene bodies has been widely reported to be positively correlated with enhanced gene expression [36, 37]. Although this is not entirely understood, it is becoming more appreciated that DNA methylation in these regions can orchestrate gene expression by suppressing spurious transcription initiation, regulating alternative splicing, ensuring correct splicing and translation, and by the recruitment of other epigenetic modifiers, such as histone methylation modifiers [38, 39]. DNA methylation is reversible in nature and methylated cytosines can be enzymatically removed by the ten‐eleven translocation proteins (TET1‐3), leading to the formation of breakdown products such as 5‐hydroxy‐methylcytosine (5H‐MeC) [40], demonstrating the dynamic and complex regulatory action on gene transcription of this epigenetic mechanism to regulate gene expression [41].

The influence of differential DNA methylation in patients with DM has been well documented in the literature. Follow‐on studies from the Diabetes Control and Complications Trial (DCCT) [42] and in the Epidemiology of Diabetes Interventions and Complications (EDIC) [43] studies highlighted persistent differential methylation at several genomic loci over more than a 16–17 years follow up period between cases and controls [44]. Of note, a significant reduction in methylation at the 3′ untranslated region of thioredoxin‐interacting protein (TXNIP) gene, known to be associated with oxidative stress and diabetic complications [45], was detected to persist in both whole blood and leukocytes (monocytes and lymphocytes) in the case cohort (patients with HbA1c > 9.1% and significant progression of retinopathy and/or nephropathy) [46]. Reduced methylation of the TXNIP gene under hyperglycaemic conditions was validated in THP1 cells and was mirrored by a marked increase in its expression [46]. Interestingly, enhanced expression of TXNIP has also been reported in the hearts of STZ‐mice (Streptozotocin‐induced diabetic models in mice), with improved glucose uptake and preserved inotropic response to β‐adrenergic receptor stimulation reported with cardiomyocyte specific deletion of TXNIP [47], suggesting a potential mechanistic involvement of DNA methylation in regulating myocardial expression of TXNIP. Similarly, enhanced expression of liver X receptor alpha (LXRα) in the diabetic rat myocardium has also been associated with hypomethylation of 9 CpGs across a 213‐bp region in the gene promoter region. Increased myocardial expression of LXRα through hypomethylation may serve as a protective response in the heart under hyperglycaemic stress, as treatment of db/db mice with an LXR agonist (GW3965) demonstrated a reduction in myocardial inflammation and collagen deposition coupled with improvements in diastolic function [48].

Enrichment in DNA methylation variations at specific regions of the genome with a linkage disequilibrium association to T2DM using whole blood samples was demonstrated by Toperoff and colleagues. This study identified significant differences in methylation at intronic regions of genes such as KCNQ1, TCF7L2, JAZF1, THADA and FTO in T2DM patients compared with healthy volunteers [49]. Interestingly, subsequent bisulfite sequencing validation revealed that a specific CpG site in the first intron of FTO is hypomethylated in T2DM subjects compared with the control group. Although the functional impact of FTO hypomethylation on its expression was not examined in this study, it has been recently demonstrated that levels of FTO in diabetic murine myocardium are significantly reduced [50]. Whether or not hypomethylation at this intronic CpG site of FTO is present in the T2DM myocardium, and its relevance to reduced myocardial FTO expression in the context of DM remains to be investigated further.

At the tissue level, DNA methylation differences have been identified in the context of hyperglycaemia and T2DM. Nilsson and colleagues conducted genome‐wide transcriptome and CpG methylation analysis in adipose tissue from patients with T2DM versus non‐diabetic subjects and found differential methylation at sites representing candidate T2DM genes, specifically IRS1, KCNQ1, TCF7L2 and PPARG [51]. Interestingly, their work highlighted a reduced expression of PPARG, an essential regulator in adipogenesis and inflammation, in the adipose tissue from discordant twins [51]. PPARG expression has been shown to be significantly elevated in the hearts of diabetic animal models [52, 53], which suggests that tissue specific changes in DNA methylation may occur and impact cellular function, metabolism and inflammation in the diabetic environment promoting tissue specific dysfunction. Within the heart itself, a recent study using methylated and hydroxymethylated DNA immunoprecipitation‐sequencing highlighted enhancement of 5MeC and 5hMeC across the genome in the hearts of STZ rat hearts that is accompanied by an increase in DNMT3B, MeCP2 and MBD2 expression [40]. Differential methylation and hydroxymethylation in the diabetic ventricular tissue were associated with pathways related to Ca^2+^, Rap1, phospholipase D, apelin and phosphatidylinositol signalling. Their work demonstrated that treatment with alpha‐ketoglutarate (a‐KG) blunted the increase in global 5MeC and 5hMeC and attenuated myocardial fibrosis and cardiomyocyte degeneration in diabetic animals. Mechanistically, the authors noted that in response to a‐KG treatment, lowered the enrichment of methylation and hydroxymethylation in the TGFBR2 and TGFBR3 intronic regions, which resulted in a reduction in their mRNA expression. This study demonstrates that reverting hyperglycaemic damage to cardiac tissue (e.g., promoting cardiac fibrosis) might be possible by reducing adverse epigenetic signatures by supplementing epigenetic modulators such as a‐KG along with an existing antidiabetic treatment regimen [40]. An additional study reported global DNA hypermethylation within the cardiac tissue in diabetic mice [54]. The level of DNA methylation increased further when the diabetic mice were subjected to ischaemic reperfusion injury, which coincided with alterations in mitochondrial functional genes [54].

Co‐repressor proteins that bind to methylated DNA have also been implicated in the pathogenesis of DCM. MeCP2 levels are significantly increased in the fibrotic myocardium of diabetic patients and STZ‐treated rats and negatively regulate Ras association domain family 1 isoform A (RASSF1A) levels to promote Ras/ERK1/2 activation and proliferation of fibroblasts in response to hyperglycaemia [55]. In addition to methylation occurring in CpG sequences of nuclear DNA, this chemical modification also takes place in mitochondrial DNA (mtDNA), regulating both mitochondrial DNA transcription and replication, termed ‘mitoepigenetics’ [56]. However, the presence of cytosine methylation in the mitochondrial genome is debated, and this requires further studies [57]. Methylation of mtDNA consists of adding a methyl group to a DNA base, usually cytosine (C) or adenine (A), from S‐Adenosyl methionine, mediated by DNMTs [56]. Importantly, compelling evidence indicates that mtDNA gene transcription is impacted by DNMTs, demonstrating that mitochondrial gene transcripts are regulated by mtDNA methylation [58]. In the heart, mtDNA methylation alteration influences mitochondrial bioenergetics and transcriptional regulatory processes involved in mitochondrial dynamics, including mitochondrial fission and fusion [59]. To underline the importance of mtDNA, studies reveal that modifications of mtDNA are associated with insulin resistance, myopathies and DM complications [60, 61, 62]. Changes in mtDNA methylation correlate with insulin resistance and the subsequent conditions of prediabetes and T2DM [63]. Besides, methylation of mtDNA in vascular smooth muscle cells contributes to impaired mitochondrial function and loss of contractile phenotype in vascular occlusive disease [64]. However, the role of mtDNA methylation in the progression of DCM has yet to be investigated. Interestingly, mtDNA is devoid of protective histones, and its close proximity to the ROS‐producing electron transport chain therefore increases its susceptibility to mutations [65]. In the context of diabetic retinopathy, it has been reported that epigenetic modifications promote a decrease in the expression of MutL homolog 1 (Mlh1), a key enzyme responsible for repairing base mismatches, leading to a decrease in mitochondrial accumulation and subsequent repair of mtDNA mismatches [66]. It is suggested that strategies targeting the regulation of DNA methylation could have the potential to protect mitochondrial homeostasis by preventing base mismatches and the development of diabetic retinopathy. It remains to be investigated whether and how this mechanism contributes to the development of DCM.

## 4 Histone Modifications

Histone modifications are small chemical modifications to the amino acid side chains of histone proteins that are added and removed by a multitude of highly specific enzymes, which influence chromatin structure to finely regulate gene expression [67]. Modification at specific positions within histone tails can affect both the histone‐DNA interactions and inter‐nucleosomal interactions, facilitating remodelling of the chromatin structure and influencing the accessibility of transcriptional machinery binding to the DNA to regulate gene expression [68]. Furthermore, it has become increasingly apparent that regulation of gene expression by histone modifications is mediated by the recruitment of effector modules or ‘readers’ to local chromatin sites that recognise the modifications and have a functional impact on transcriptional activity [69]. Modifications to histone proteins that are known to influence gene expression include acetylation, methylation, phosphorylation, poly‐ADP‐ribosylation, ubiquitination, deamination, sumoylation and glycosylation [70].

The best characterised of these modifications are lysine acetylation and deacetylation, and lysine methylation and demethylation. Histone acetylation is the process by which acetyl groups are added to ε‐amino group of lysine side chains and is catalysed by the histone acetyltransferase enzymes (such as GNAT family, p300, The MYST family) [71]. Addition of acetyl groups results in neutralisation of the charge interaction between the negatively charged DNA backbone and the positively charged lysine residues, promoting relaxed chromatin conformation and active gene transcription. Removal of acetyl groups is carried out by histone deacetylase enzymes (HDACs), which result in chromatin condensation and, therefore, repress transcriptional competence [72].

The process and consequences of histone methylation are more complex than acetylation. Lysine residues on histone tails can be methylated to different degrees (mono‐, di‐ or tri‐methyl) by methyltransferase enzymes (e.g., SET‐domain family and the DOT1L/KMT4 family) and subsequently removed under the action of histone demethylase enzymes LSD demethylases (e.g., LSD1 and LSD2) and the Jumonji C‐domain containing demethylases (JMJDs). Methylation at specific lysine residues on histone protein 3, H3K4 (enrichment of H3K4me1 at enhancer elements and H3K4me3 at active promoters), H3K26 and H3K79 are associated with active gene transcription, whereas methylation at H3K27, H3K9 and H3K20 is associated with transcriptionally silenced heterochromatin [73]. Protein complexes can also modify chromatin structure through regulation of histone methylation activity, such as the Trithorax group (promotes gene transcription through their H3K4 trimethylase activity) and the silencing polycomb group (enhancer of zeste homolog 2 (EZH2) has H3K27‐specific trimethylase activity) complexes, both of which have been shown to be critical for normal cardiac development and function [74].

Mounting evidence has highlighted the contribution of dysfunctional changes in both histone acetylation and methylation in the failing heart [75, 76], with several studies describing alterations in the context of DCM. Changes in both histone acetylation and methylation have been reported to influence cardiac cell function under hyperglycaemic conditions. Culture of cardiomyoblasts (H9c2 cells) in high glucose resulted in enhanced cellular apoptosis found to be mediated by the reduction in IGFR1 expression associated with HDAC1 recruitment by p53 and lower levels of acetylation at the IGF1R promoter region [77]. Exposure of cardiomyoblasts to high glucose levels was also found to enhance the expression of IL‐6, even when cells were returned to physiological conditions. This maintained pro‐inflammatory response was associated with reduced expression of the SET‐domain methyltransferase Suv39h1 and reduced H3K9me3 marks at the IL‐6 promoter [78]. Subsequent establishment of a pro‐inflammatory cellular phenotype by hyperglycaemia‐induced changes in histone methylation was also demonstrated in human endothelial cells. Transient exposure of endothelial cells to high glucose levels promoted a Set7‐mediated increase in H3K4me1 marks at the promoter region of p65 that resulted in augmented expression of pro‐inflammatory mediators VCAM‐1 and MCP‐1, even after subsequent culture at physiological glucose levels [79, 80]. Moreover, Set7 methylation of H3K4 impacted cardiac structure, function, and metabolic parameters in response to chronic stresses, such as isoproterenol‐induced cardiac hypertrophy, epigenetic hyperglycaemic memory, and glucose tolerance [81]. Targeting Set 7 may therefore serve as a therapeutic treatment in response to chronic stress, including metabolic disturbances [82, 83].

Various studies utilising different preclinical models of DM have indicated alterations in both histone methylation and acetylation associated with pathological cardiac remodelling in vivo. Global changes in H3 histone marks reduced H3K9me2 and increased acetylation (H3K23 and H3K9), and H3K4me2 were found in cardiac tissue of db/db mice with renal complications [84]. In response to STZ‐induced hyperglycaemia in CD1 mice, increased nuclear H3K27me3 accompanied by a reduction in H3K9 acetylation, was measured in cardiac tissue after 12 weeks post‐STZ administration [85]. In spontaneously diabetic Goto‐Kakizaki rats with pronounced post‐ischaemic cardiac remodelling induced by myocardial infarction, cardiac Sirt‐1 levels (class III HDAC) were significantly upregulated and accompanied by a reduction in levels of acetylated‐p53 in the remote myocardium [86]. The associated increased expression of Sirt‐1 is postulated to be a cardioprotective response to hyperglycaemic injury, as pharmacological activation of Sirt‐1 resulted in attenuation of cardiac hypertrophy and oxidative stress through a reduction in acetylation of NF‐kB‐p65 and global cardiac H3K9 levels in fructose‐fed diabetic rats [87]. Other pharmacological intervention studies targeting HDACs have also demonstrated therapeutic benefits in the context of DCM. This includes evidence that HDAC inhibition ameliorates cardiac performance and mitigates metabolic disturbances in murine models of both type I and type II diabetes [76]. For example, administration of a selective HDAC3 inhibitor RGFP966 prevented DCM progression in diabetic OVE26 mice, with treated animals, through a sustained reduction in inflammation, oxidative stress and cardiac fibrosis. HDAC3 inhibition resulted in enhanced acetylation at the promoter region of the nuclear phosphatase DUSP5 gene, leading to increased expression and its inhibitory action on ERK1/2 activity [88]. Moreover, Hu and colleagues [89] demonstrated that PRDM16 exhibited protection of myocardial lipid metabolism and mitochondrial function in DCM mice depending on epigenetic regulation of H3K4me3 by modulating PPAR‐α and PGC‐1α. Therefore, developing a PRDM16 activator targeting PPAR‐α and PGC‐1α pathways could be a novel therapeutic strategy for treating cardiac abnormalities in T2DM [89].

## 5 BET Proteins

**FIGURE 3:**
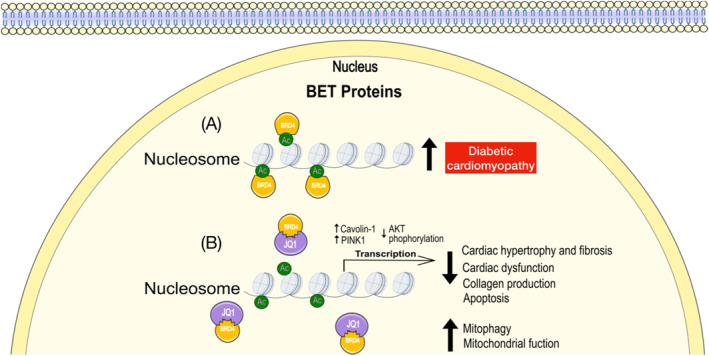
Bromodomain and extra‐terminal domain (BET) proteins and diabetic cardiomyopathy. (A) BRD4 is a member of the BET family of epigenetic regulators and is upregulated in diabetic cardiomyopathy, resulting in accumulation of damaged mitochondria and subsequent impairment of cardiac structure and function. (B) BET inhibitor (JQ1) alleviates the pathogenesis of diabetic cardiomyopathy with improved mitochondrial function and repaired cardiac structure and function. BET = Bromodomain and extra‐terminal domain; BRD4 = Bromodomain‐containing protein 4.

The bromodomain and extra‐terminal domain (BET) family of proteins (BRD2, BRD3, BRD4 and BRDT) function as epigenetic reader proteins that are now appreciated to play an essential role in gene regulation in both physiological and pathological states. These proteins mediate their regulatory function by coupling proximal signals and multi‐protein complexes to transcription factors and chromatin marks (e.g., acetylated histone marks) [90]. Mounting evidence suggests that BET proteins are key elements underlying the pathophysiology of CV complications [91, 92], with reports demonstrating their alterations in response to hyperglycaemia. Among BET proteins, BRD4 is the most well‐researched BET protein, known as the ‘reader’ of lysine acetylation in the context of response to various stressed conditions, including the diabetic heart [93, 94]. BRD4 expression has been shown to be significantly increased in both cardiomyoblasts [95, 96] and fibroblasts [95] exposed to high concentrations of glucose in vitro. In support of the aberrant role in hyperglycaemia, elevated expression of BRD4 was demonstrated in the myocardium of several preclinical models of DCM (STZ‐mice and rats and HFD‐mice), with pharmacological inhibition of BET activity (BET inhibitor, JQ1) leading to attenuation of pathological cardiac remodelling and subsequent improvements in cardiac function [94, 95, 96]. Reported mechanistic benefits of inhibiting the action of BRD4 in hyperglycaemia conditions are associated with increased caveolin‐1 expression [95], reduced AKT phosphorylation [96] and most recently, activation of PINK1/Parkin‐mediated mitophagy [94] (Figure 3).

Although primarily under investigation in clinical oncology, the clinical utility of selective BET inhibitors for the treatment of CV disease has recently been examined in the recent Phase 3 BETonMACE trial. Unfortunately, treatment with the bromodomain 2 (BD2)‐specific BET inhibitor apabetalone showed no significant difference in major adverse CV events (CV death, stroke and myocardial infarction) in T2DM patients with recent acute coronary syndromes and low HDL‐c compared with placebo [97]. However, exploratory analyses of this trial have suggested that apabetalone may have CV benefits in specific at‐risk populations. Patients with T2DM, recent acute coronary disease, and a moderate likelihood of Non‐alcoholic Fatty Liver Disease were found to have a significantly lower rate of ischaemic adverse CV events and hospitalisation for heart failure after treatment with apabetalone [98]. Similarly, a reduction in hospitalisation for heart failure was also noted in T2DM patients with chronic kidney disease and lower LDL‐C levels who received apabetalone compared to placebo [99]. While further in‐depth analysis from the BETonMACE trial is still ongoing, BET inhibition in T2DM patients seems to show therapeutic impact on CV events and highlights the potential for specifically targeting other BET proteins, such as BRD4, that may prove to be an effective therapeutic strategy for patients with DCM.

## 6 microRNAs

**FIGURE 4:**
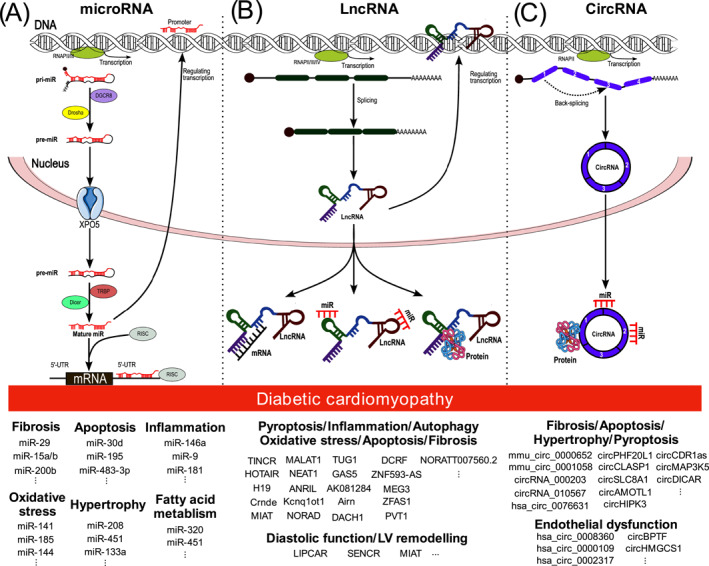
Biogenesis and function of non‐coding RNAs (miR, lncRNA, circRNA) and diabetic cardiomyopathy. (A) microRNAs (miRs) are initially transcribed as primary miRs (pri‐miRs), which have a distinct stem‐loop structure. These pri‐miRs are processed in the nucleus by Drosha and DGCR8 (also called Pasha) into precursor miRs (pre‐miRs). The pre‐miRs are then transported to the cytoplasm via Exportin 5, where they are cleaved by Dicer into a miRNA duplex. One strand of the miRs duplex is integrated into the part of the miRNA‐induced silencing complex (RISC), and the second strand is degraded. The RISC complex binds to target mRNAs through base‐pairing between the miRs and the mRNAs, leading to either suppression of translation or degradation of the mRNA. Moreover, there are unconventional/atypical miR functions such as activation of Toll‐like receptors (TLRs). (B) Most long non‐coding RNAs (lncRNAs) are processed similarly to mRNAs, involving capping, splicing, and adenylation, with some exceptions. Mature lncRNAs form complex 3D structures that enable their diverse functions, which vary by cellular location. Chromatin‐bound lncRNAs (often cis functions) typically regulate transcription, chromosome looping, and histone modifications. Cytoplasmic lncRNAs bind mRNAs, acting as decoys, guides, and scaffolds to regulate genes transcriptionally or post‐transcriptionally. They also bind proteins to alter their function and stability, code for micropeptides that are being translated, and bind other non‐coding RNAs, including miRs. (C) CircRNAs have multiple biogenesis mechanisms, but back‐splicing is a common event for all circRNAs. circRNA molecules are rich in miR‐binding sites and act as miR sponges in cells, thereby lifting the inhibition of miR on target genes and increasing the expression level of target genes. Additionally, circRNAs can also interact with proteins.

microRNAs (miRs) are RNA molecules comprised of 18–22 nucleotides that regulate gene expression at the level of mRNA translation by binding to mRNAs and targeting them for repression or degradation. Transcriptional regulation by miRs is complex as individual miRs can control the expression of numerous target mRNAs, and each target mRNA can be regulated by multiple different miRs [100]. Considering their key roles in modulating the expression of multiple genes, it is no surprise that several candidate miRs have been highlighted in clinical samples and various preclinical diabetic models to be involved in the numerous pathophysiological processes in DCM, for instance, excessive oxidative stress (miR‐144 [101], miR‐14 [102], miR‐185 [103], miR‐673 [34]), fatty acid metabolism (miR‐320 [104]), fibrosis (miR‐29 [105], miR‐15a/b [106], miR‐200b [107]), apoptosis (miR‐30d [108], miR‐195 [109], miR‐483‐3p [110]), myocyte hypertrophy (miR‐208a [111], miR‐133a [112], miR‐451 [113]) and inflammation (miR‐146a [114], miR‐9 [115], miR‐181 [116]). In diabetic hearts, specific miR expression, such as miR‐1 was found to be upregulated [117] or downregulated [118], suggesting that the specific miR could respond differently to the trigger based on complexities and involve multiple mechanisms in the progression of DCM. Furthermore, overexpression of miR‐200a‐3p in DCM mice mitigated cardiac dysfunction, fibrosis, inflammation, cardiomyocyte apoptosis and increased autophagy [119]. miR‐494 was recently reported to upregulate expression in DCM rats and was associated with cell apoptosis and autophagy [120]. Therefore, augmenting miR‐200a‐3p and inhibiting miR‐494 may provide therapeutic effects in the context of DCM [119, 120]. These results underline the complex regulation of the specific miR in response to determinants and potentially promote multiple pathogenic drivers of DCM. It also seems that changes in miR expression in response to hyperglycaemic insult may persist even after glycaemic levels return to normal, facilitating pathological development in the heart [121]. Conversely, recent reports have suggested that miR expression may be positively changed through interventional methods, such as high‐intensity exercise training, to reduce associated cardiac dysfunction with diabetes [122]. The expression of many miRs is dysregulated in the diabetic heart and may be modified in the plasma of diabetic individuals. For this reason, miRs have been proposed as potential biomarkers for the prognosis and diagnosis of patients with DCM. To enhance the understanding of the complexity of miRs involved in DCM progression, the DCM‐related miR‐mRNA network and DCM‐related competitive endogenous RNA (ceRNA) were investigated [123]. The results identified five hub genes—phospholamban (Pln), fatty acid binding protein 3 (Fabp3), tripartite motif‐containing protein 63 (Trim63), popeye domain containing 2 (Popdc2), and troponin C1 (Tnnc1)—related to ceRNA, which are accountable for the onset of DCM [123]. The involvement of miRs in the pathogenesis of DCM, as well as in the context of potential therapeutic targets, have been comprehensively summarised elsewhere [124, 125, 126, 127, 128, 129, 130, 131] (Figure 4).

## 7 LncRNAs

Long non‐coding RNAs (lncRNAs) are diverse RNA transcripts composed of > 200 nucleotides that are now appreciated to possess numerous gene regulatory roles through their interaction with diverse macromolecules, including other RNA species, proteins, and DNA [132]. As a result, dysfunctional changes in the expression of these lncRNA molecules in the pathogenesis of heart failure are under intense investigation, with myriad lncRNAs identified to be aberrantly expressed in the diabetic myocardium [133]. The study by Pant and colleagues conducted a comprehensive analysis in 20‐week‐old db/db mice with diastolic dysfunction and identified 754 lncRNAs that were differentially regulated compared to healthy control mice [134]. Co‐expression network analysis revealed 5 lncRNAs (BC038927, G730013B05Rik, 2700054A10Rik, AK089884, and Daw1) that were connected with several differentially expressed genes associated with membrane depolarisation, action potential conduction, contraction, and actin filament‐based movement of cardiac cells in the diabetic myocardium [134]. Moreover, methyltransferase‐like 14 (METTL14) expression was downregulated in DCM, mediated increased terminal differentiation‐induced lncRNA (TINCR), leading to pyroptosis processes via m6A methylation in an NLRP3‐dependent manner [135]. LncRNA TUG1 involved myocardial fibrosis in DCM development through modulating miR‐145a‐5p/Cfl2 axis, and suppression of lncRNA TUG1 demonstrated improvement of cardiac function and fibrosis [136]. Other key lncRNAs which have also been demonstrated in the myocardium and circulation in patients and preclinical models of DCM are HOTAIR [137], H19 [138, 139], MALAT1 [140], MIAT [141, 142, 143], SENCR [141], ANRIL [144] and LIPCAR [141] (Figure 4).

## 8 Circular RNAs

Circular RNAs (circRNAs) are single‐stranded RNA loops generated via back‐splicing events that do not possess either 5′ end caps or 3′ end poly‐A tails [145]. CircRNAs accumulate intracellularly due to their structural resistance to endonucleases and can competitively interact with other ncRNA molecules (e.g., miRs) and also regulate different aspects of RNA, including transcription, splicing, turnover and translation [145]. Several groups have identified a large number of circRNAs expressed in human and rodent hearts [146, 147], some of which are now appreciated to play an important role in maladaptive cardiac remodelling and heart failure development [148]. A study by Dong and colleagues [149] has identified and validated 13 circRNAs differentially expressed in the myocardium of diabetic BKS‐db/db knock‐out mice, with related mRNAs associated with glucose metabolism and lipid metabolism pathways. Specifically, two circRNAs, mmu_circ_0000652 and mmu_circ_0001058, demonstrated a reduction in expression in the early stage of DCM progression and were identified as potential miR sponges to miR‐195 and miR‐2, resulting in cardiomyocytes apoptosis and interstitial fibrosis, respectively [149]. Moreover, other circRNAs have been implicated in promoting pathological cardiac dysfunction in DCM through their competitive interaction with miRs. CircRNA_000203, a circRNA transcribed from the Myo9a gene, was significantly elevated in the myocardium of db/db mice and promoted pro‐fibrotic gene expression in murine cardiac fibroblasts through its inhibitory action on miR‐26b‐5p [150]. Similarly, circRNA_010567 was also significantly upregulated in db/db diabetic myocardium and was determined to competitively inhibit the action of miR‐144 on TGFβ1‐activation of cardiac fibroblasts [151]. CircDICAR (the diabetes‐induced circulation‐associated circular RNA) showed downregulation in diabetic db/db mice and diabetic patients, promoting cardiac dysfunction, hypertrophy, fibrosis, and cardiomyocyte pyroptosis, which are the pathogenesis of DCM. Thus, circDICAR and the synthetic‐junction part may rescue diabetes‐induced cardiac impairments [152]. In a recent study, alterations of multiple circRNAs such as circPHF20L1, circCLASP1, and circSLC8A1 played significant roles in underlying cardiac fibrosis in db/db mice [153].

In STZ‐induced diabetic mouse models, cardiac fibrosis is evident in DCM progression, and upregulation of circAMOTL1 and circHIPK3 in diabetic mice was linked to myocardial fibrosis. Inhibition of these circRNAs ameliorated cardiac function and fibrosis, providing a therapeutic target for DCM [154, 155]. CircRNA cerebellar degeneration‐related protein 1 antisense (CDR1as) was upregulated in the DCM setting and cardiomyocytes treated with high glucose, and this alteration promoted cardiomyocytes apoptosis via hippo signalling pathway, which is one of the pathogenic drivers of DCM [156]. Furthermore, circRNAs were reported to modulate the pathogenesis of DCM at a cellular level; levels of the circRNA hsa_circ_0076631 (also known as caspase‐1‐associated circRNA, CACR) were found to be increased in cardiomyocytes (AC16 cells) exposed to high glucose and promoted cell death and inflammation through increasing the expression of caspase‐1 by antagonising the action of miR‐214‐3p [157]. CircRNA mitogen‐activated protein kinase 5 (circMAP3K5) was increased in cardiomyocytes treated with high glucose conditions, promoting apoptosis processes in part of DCM progression through regulating the miR‐22‐3p/DAPK2 axis [158]. Others studied diabetes‐induced impairment of endothelial function. Alterations of circRNAs (e.g., hsa_circ_0008360, hsa_circ_0000109, hsa_circ_0002317, CircBPTF, and circHMGCS1) are associated with endothelial dysfunction in the context of HUVECs treated with high glucose conditions, and these results suggest that circRNAs considerably contribute to the initiation and development of DCM [159, 160, 161] (Figure 4).

## 9 Cell Type Specific Epigenetic Changes in Diabetic Cardiomyopathy

Different cardiac cells have different epigenomic signatures, and understanding cell type‐specific roles of epigenetic modification is an important issue in designing epigenetic interventions for treatment [162]. Hyperglycaemia induces epigenetic changes with the expression of genes associated with endothelial dysfunction and alteration of immune cells, including macrophages, dendritic cells, mast cells, T‐cells and B‐cells. Endothelial cells are the target of early hyperglycaemia‐induced changes with epigenetic modulation and long‐term effects. Modifications of endothelial cells are key to the development of vascular alterations in DCM, including microvessels [163] with diffuse alteration of myocardial perfusion in cardiomyopathy and later on the development of atherosclerosis with macrovascular alterations [31, 164]. The role of Set7, a histone methyl transferase responsible for the monomethylation of lysine 4 on histone 3 (H3K4) in glucose‐induced inflammation of endothelial cells, has been pointed out as a major actor in endothelial dysfunction [165]. Set7 regulates the transcription factor NFκB and contributes to the dysregulation of oxidant/inflammatory genes and endothelial dysfunction. Pepin et al. [166] have quantified genome‐wide DNA methylation of cultured human endothelial aortic cells and have investigated glucose‐dependent and dose‐responsive alterations in endothelial DNA methylation. They have shown glucose‐dependent methylation and gene expression of VEGF and NO signalling cascades, which could be involved in the long‐term effects of hyperglycaemia on endothelial function. The role of energy metabolism on epigenetic regulation in endothelial dysfunction has been explored as key energy metabolites typically serve as substrates or co‐factors for epigenetic modifying enzymes. In particular, the role of dysregulation of 2‐oxoglutarate‐dependent dioxygenases by hyperglycaemia has been suggested [167]. Besides this vascular role, the endothelium controls cardiomyocyte metabolism and function and mediates the transcellular flux from blood to perivascular tissues, which may be impaired by epigenetic modifications [168].

The endothelial‐to‐mesenchymal transition (EndMT) has been recently recognised as a major process in diabetic complications, particularly in cardiomyopathy with hyperglycaemia as an important trigger. The importance of EndMT in the pathogenesis of DCM is observed in diabetic human hearts, and this process illustrated upregulation of lncRNAs ZFAS1 and MALAT1 while decreasing several miRs (miR‐9, miR‐146a, and miR‐200b) in diabetic patients compared with non‐diabetic patients [169]. In response to different stimuli, endothelial cells are losing endothelial characteristics and developing mesenchymal traits [170]. These modifications result in cardiac fibrosis and remodelling. Epigenetic changes occur at different levels during EndMT in pathogenesis related to DCM progression involving the deacetylase HDAC3a [171], DNA methylation and methylation of RASAL1 promoter [172, 173], and miRs (miR‐126‐3p, miR‐146a, miR‐9) [114, 115, 174]. LncRNAs are also involved, especially ANRIL, which regulates functional and structural myocardial alterations in diabetes through expressions of ECM proteins and VEGF [144] and ZFAS1 interacting with miR‐9 in mediating cardiac fibrosis in human cardiac microvascular endothelial cells [115] and circRNA [175].

The cardiac fibroblast is one of the key participants in alterations of ECM proteins, causing cardiac stiffness and decreased myocardial relaxation, resulting in progressive impaired cardiac function and structure [176]. Epigenetic responses in cardiac fibroblasts treated with high glucose are extensively investigated. DNMT1 expression was upregulated and subsequently suppressed the SOCS3 axis in cardiac fibroblasts treated with high glucose conditions, resulting in cardiac fibrosis through cardiac fibroblast activation [177]. Also, DNMT1 inactivation in androgen receptor axis promoted cardiac fibroblast autophagy in diabetic cardiac fibrosis [178]. miR‐150‐5p expression was upregulated and Smad7 expression decreased in cardiac fibroblasts treated with high glucose, driving cardiac inflammation and fibrosis [179]. The study by Liu et al. [180] also showed that elevated miR‐21 promoted proliferation and collagen production in rat cardiac fibroblasts treated with high glucose via the JNK/SAPK and p38 signalling pathways by repressing DUSP8 expression. Besides, increased miR‐32‐5p could repress DUSP1, contributing to cardiac fibrosis in the context of human cardiac fibroblasts in response to high glucose stimulation [181]. Conversely, miR‐495 demonstrated protective effects against inflammation, cell differentiation, and ECM accumulation in high glucose‐activated NF‐κB and TGF‐β1/Smad signalling pathways in human cardiac fibroblasts [182]. Elevated lncRNAs such as Kcnq1ot1 and AK081284 in cardiac fibroblasts treated with high glucose contributed to cardiac fibrosis via pro‐fibrotic pathways [183, 184], while increased lncRNA Airn mitigated cardiac fibrosis by inhibiting activation of cardiac fibroblasts via the m6A‐IMP2‐p53 axis [185]. These results underline the significant roles of epigenetic modifications on fibroblasts in impacting DCM progression.

Long‐term hyperglycaemia conditions may contribute to alternations of cardiomyocyte calcium handling, hypertrophy, autophagy, and apoptosis, which are important pathophysiological processes of DCM [186]. Under high glucose conditions, multiple miRs‐associated with cardiomyocyte hypertrophy, such as miR‐30c, miR‐133a, miR‐150, miR‐373, and miR‐203, were downregulated [112, 187, 188, 189, 190], whereas miR‐451 was increased in the diabetic heart [113]. The transcriptional co‐activator p300, histone acetyltransferase, participates in cardiomyocyte hypertrophy in the context of hyperglycaemia [187]. In addition, DCM‐related to cardiomyocyte apoptosis and mitochondria dysfunction are revealed through alterations of miRs, including miR‐34a [191], miR‐1 [192, 193], miR‐206 [192], mi‐195 [194] and mi‐30d [108]. lncRNA H19 showed inhibition effects on autophagy of cardiomyocytes and cardiomyocyte apoptosis [138, 139]. Recently, knockdown of lncRNA GAS5 improved high glucose‐induced cardiomyocyte inflammation, apoptosis, and oxidative stress injury [195, 196]. Other epigenetic modifications, such as BET proteins (BRD4) in cardiomyocytes in the context of DCM, have previously been reviewed [94, 130].

Moreover, the global methylation status of leukocytes and B‐cells has been shown in obesity and diabetes [197, 198]. DNA methyltransferase 3B (DNMT3B) is increased in macrophages exposed to high levels of saturated fatty acids and promotes M1 macrophage polarisation [199]. Histone deacetylases (HDACs) are important in controlling inflammation; for instance, HDAC3 regulates inflammatory genes in macrophages, and HDAC2 contributes to the resolution of inflammation [200]. Numerous miRs are involved in immune cells, such as miR‐146b, resulting in monocyte activation, miR‐107 inducing increased macrophage responses, miR‐126 and miR‐193b, resulting in enhanced chemotaxis [201].

## 10 Epigenetic Cooperation

Although addressed individually in this review, it is well appreciated that epigenetic modifications not only act individually but can collectively cooperate with one another to dynamically modulate chromatin structure and gene expression in the diabetic heart. A recent study has highlighted the involvement of DNA methylation, histone modifications and miRs to regulate the expression of JunD, a member of the activated protein‐1 family that plays an important protective role against oxidative stress, inflammation and remodelling [34]. The reduction in JunD expression in the diabetic myocardium was underlined by the presence of increased DNA methylation and repressive histone marks, along with a reduction in active histone marks at the JunD promoter. Furthermore, cardiac expression of miR‐673 was reduced in the diabetic murine heart, leading to upregulation of Men1, a negative regulator of JunD activity and a known recruiter and component of histone methylation complexes, highlighting the dynamic interplay of epigenetic modifiers in regulating target gene expression in metabolic dysfunction in DCM [34]. To support targeting multiple epigenetic alterations related to DCM, flavonoids, natural epigenetic modulators, can modulate epigenetic networks with multiple mechanisms, including DNA methylation, histone modifications, ncRNA‐based modifications, and chromatin remodelling, leading to prevention of DCM development. For example, quercetin demonstrates cardioprotective benefits in diabetic hearts through modification of DNA methylation and HDAC4 inhibition [202, 203, 204]. Ultimately, as these epigenetic modifications are reversible and pharmacologically targetable, the concept of epigenetic therapy is currently being explored as a potential treatment strategy for DCM; however, further research is required to elucidate the contribution and cooperation of these underlying mechanisms to influence stable and hereditable pathological cellular changes that mediate diabetes‐induced dysfunction in the heart.

## 11 Epigenetic Biomarkers and Diabetic Cardiomyopathy

Early detection and management of DCM are crucial to prevent its progression to heart failure. Epigenetic biomarkers have emerged as promising tools for understanding the molecular mechanisms underlying DCM development and identifying individuals at risk. Due to their reported stability in various human fluids and their ability to be detected quantitatively, epigenetic markers have been suggested as potential diagnostic biomarkers for CV diseases such as myocardial infarction, coronary artery disease, and heart failure [124, 205]. With regard to DCM, the primary associated epigenetic modifications are DNA methylation and histone modifications [206]. Changes in DNA methylation patterns of specific genes, including those involved in insulin signalling and inflammation, have been linked to DCM [207]. Aberrant histone acetylation and methylation have also been observed in DCM, affecting chromatin structure and gene expression [208]. Tao and colleagues [209] observed increased expression of methyl CpG binding protein MeCP2 and DNA hypermethylation in the RAASF1A promoter region in patients with DCM and in a T1DM rat model. These data suggest that MeCP2 plays a role in the regulation of DCM and could be a potential marker to predict DCM. It has also been reported that HiF3A mRNA expression in peripheral blood is decreased in DCM patients, which was associated with highly methylated CpG sites on HIF3A intron 1. This was consistent in patients with DCM compared with those with T2DM, suggesting that HIF3A intron 1 methylation at CpG6 could be used to identify patients with DCM from those with T2DM [210]. In addition, several groups have observed and suggested an association of hypomethylation of the KEAP1 promoter, decreased levels of H3K9me3 and increased levels of DNMT, which enhanced methylation of SERCA2a, thereby decreasing SERCA2a expression in patients with DCM [29].

While DNA methylation and histone modifications have been reported as primary epigenetic changes associated with DCM, studies have suggested that circulating miRs can also be altered depending on the phase of disease; therefore, in terms of evaluating the development and progression of DCM, they could be used as potential biomarkers [207]. Marked molecular changes in the heart caused by miRs have been associated with diabetes, and these changes appear to occur prior to any structural and functional changes. Additionally, the synthesis of proteins needed for normal cardiac function is inhibited in diabetic hearts by altered expression of miRs. As they are also released into the bloodstream, these CV miRs can be readily detected as biomarkers [205]. Although there have been no associations with specific miRs in patients with DCM, it is likely that circulating miRs observed in diabetes may also be associated with DCM [124]. The associated miRs which could serve as potential serum biomarkers for DCM include miR‐9, miR‐21, miR‐29, miR‐30d, miR‐34a, miR‐144, miR‐150, miR‐320 and miR‐378 [124]. Additionally, Copier et al. [211] observed an association between circulating miR‐19b‐3p and miR‐181b‐5p levels and myocardium levels during the development of DCM in obese mice on HFD. These data suggest that miR‐19b‐3p and miR‐181b‐5p could potentially serve as biomarkers for the progression of DCM in patients. While the beginning of the ‘big data’ era was marked by the accumulation of dataset integration, high‐throughput sequencing, omics‐based approaches and deep sequencing, advanced network‐based algorithms and Artificial Intelligence (e.g., machine learning) approaches may facilitate the prioritisation of molecules with functional relevance for preventing and treating the CV risk in individuals with diabetes [212]. Gadd et al. [213] developed a machine learning‐based epigenetic scoring tool using DNA methylation data to capture methylation‐proteomic signatures associated with diabetes, enabling prediction and risk stratification. This tool could hold potential for identifying diabetes‐associated CV complications. Furthermore, integrating physiological, biochemical, genetic, and epigenetic features with machine learning algorithms may improve the diagnosis and understanding of diabetes‐induced CV dysfunction. Hathaway et al. [214] identified novel cardiac biomarkers and genomic features linked to T2DM, showing that gene promoter methylation changes can serve as predictive markers for pathological processes in DCM.

Altogether, epigenetic biomarkers hold promise for early diagnosis, risk stratification, and therapeutic targeting of DCM. However, further research is needed to validate these biomarkers in large patient cohorts and to understand their functional significance in disease progression.

## 12 Epitranscriptomics and Diabetic Cardiomyopathy

While epigenetics has become relatively well known in the cardiovascular research field, the field of epitranscriptomics has been less explored. Similar to epigenetics, epitranscriptomics relates to the study of nucleotide modifications but at the level of RNA. RNA modifications can be chemical, at the level of alternative splicing, or through changes in base composition (‘RNA editing’). N6‐methyladenosine (m6A) RNA modification is the most prevalent and better characterised chemical modification of RNA. Its role in cardiovascular development and disease starts to be relatively well characterised [215]. Alterations in m6A profiles are associated with diabetic microvascular dysfunction [216]. Technical advances as well as the development of bioinformatics tools allow improving the knowledge of the role of RNA editing in cardiovascular disease [217]. Aberrant splicing of the CaV1.2 calcium channel mediated by the RNA‐binding protein RBFOX2 has been reported in diabetic rats developing diastolic dysfunction and cardiac hypertrophy [218, 219]. Alterations and functional associations between RNA editing, splicing and DCM development remain overall poorly explored.

## 13 Future Perspectives and Conclusions

Epigenetics is a promising field, and its modifications are related to diabetes‐induced cardiac impairments. Targeting the epigenome might be a new paradigm treatment option for treating or slowing down DCM development. As the progression of DCM is unique and progressive, with distinction from other forms, epigenetic regulation plays a fundamental role in the pathogenesis of DCM. Therefore, epidrugs represent a plausible therapeutic approach to target manifestation and progression in the context of DCM. While numerous preclinical studies have shown that certain molecules have therapeutic potential, clinical trials are necessary to assess their pharmacokinetic and pharmacodynamic characteristics, safety profiles, appropriate dosages, and delivery methods. Evidence is mounting on BRD inhibitors in DCM treatments, but these studies to date are primarily from animal models [94, 95]. Supporting this is the finding that treatment with the BRD inhibitor‐apabetalone in patients with T2DM and recent acute coronary syndrome was associated with reduced hospitalisation for heart failure [220]. There is biological plausibility of BRD inhibitors for DCM treatment by modulating DCM pathogenesis [94, 95], and clinical trials to directly assess their effects on manifestation and progression of DCM are important. This translational approach should be considered more widely given that the majority of DCM studies to date are preclinical in nature. Greater efforts towards clinical translation are needed whilst being mindful of limitations with species conservation and preclinical models that do not fully reflect human pathogenesis. When considering the translation of epigenetic markers from preclinical studies to clinical phase, such as using lncRNAs as epigenetic drug targets or biomarkers, it is important to note that most lncRNAs are less conserved among species compared with protein‐coding mRNAs [221]. For example, the lncRNA HOTAIR was found to be associated with DCM in preclinical models [137]; however, caution is needed when translating this finding to human studies as structural and functional differences of lncRNA HOTAIR between mouse and human have been observed, indicating potential species‐specific variation in lncRNA function [222]. These highlight the importance of considering differences among species in extrapolating findings from DCM preclinical models to human biology. Moreover, screening for DCM based on epigenetic blood‐based biomarkers could be useful for identifying patients at risk of developing DCM and is considered a proactive strategy. There are currently no approved epigenetic blood‐based biomarkers for DCM diagnosis. One of the potential epigenetic blood‐based biomarker candidates for DCM is circulating miRs, which are altered in diabetes associated with cardiomyopathy and may play a pivotal role in the pathology of DCM. These miRs, including miR‐9, miR‐21, miR‐29, miR‐30d, miR‐34a, miR‐144, miR‐150, miR‐320, and miR‐378, are highly related to DCM [124]. In line with this, a clinical study by Tao et al. [223] demonstrated that circulating miR‐21 serves as a novel biomarker for the diagnosis of DCM and offers mechanistic insights supporting the potential of miRNA‐based therapeutic strategies for DCM treatment. In the future, cardio‐specific epidrugs using specific delivery methods may serve to advance therapeutic development to epigenetically target DCM. Rapid scientific research methods and technologies such as single‐cell RNA sequencing (scRNA‐seq), single‐cell assay for transposase‐accessible chromatin sequencing (scATAC‐seq), and whole genome bisulfite sequencing (WGBS) potentially support the discovery of new epigenetic modifications. With the recent development of direct RNA sequencing and other mass spectrometry‐based technologies, epitranscriptomics has entered the game, and the regulation and roles of RNA modifications, editing, and splicing in DCM deserve further investigation. Altogether, understanding the progression of DCM through epigenetic and epitranscriptomic alterations and developing novel therapies based on these alterations to prevent the onset or progression of DCM are likely on the horizon. Biomarkers and treatments based on epigenetic or epitranscriptomic marks may be useful for developing ‘theranostics’ approaches [224].

**FIGURE 5:**
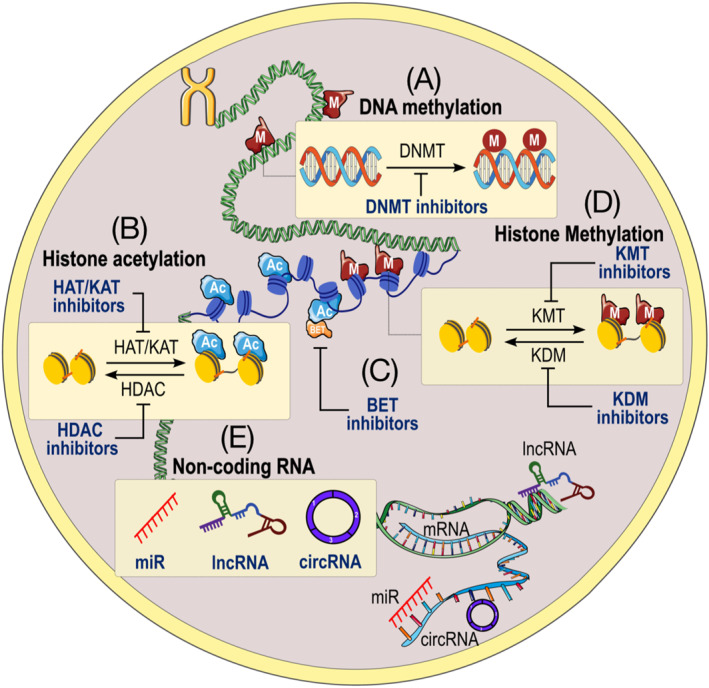
Potential epigenetic targets in diabetic cardiomyopathy. (A) DNA methylation occurs predominantly at CpG islands, and DNMT inhibitors can block the activity of DNMTs, enzymes responsible for adding methyl groups to DNA. (B) Histone acetylation depends on the writer HAT/KAT, the reader acetyl‐lysine binding proteins, and the eraser HDAC. HAT/KAT inhibitors are compounds that block the activity of acetyltransferases, which are responsible for adding acetyl groups to histone proteins, affecting DNA packaging and gene expression. HDAC inhibitors exhibit histone deacetylase inhibition by preventing HDAC from removing acetyl groups from histones. (C) BET proteins can bind to acetylated histone tails, and BET inhibitors reversibly bind BET proteins and prevent protein‐protein interaction between BET proteins and acetylated histones. (D) Histone methylation involves the addition of methyl groups to histone proteins, and KMT inhibitors block histone lysine methyltransferases (KMT), and KDM inhibitors inhibit histone lysine demethylases (KDM). (E) Non‐coding RNAs and alterations of miR, lncRNA, and circRNA. These non‐coding RNAs are functional RNA molecules that are not translated into proteins, playing crucial roles in regulating biological responses. BET = Bromodomain and extra‐terminal domain; circRNA = Circular RNA; DNMT = Deoxyribonucleic acid methyltransferase; HAT = Histone acetyltransferase; HDAC = Histone deacetylase; KAT = Lysine acetyltransferases; KDM = Lysine demethylases; KMT = Lysine methyltransferase; lncRNA = Long non‐coding RNA; miR = MicroRNA.

In summary, DCM is an important stage which is responsible for future heart failure development. Epigenetic alterations, including DNA methylation, histone modifications, and ncRNAs, can regulate multiple pathogenic drivers of DCM. Therefore, the epigenetic approach is a visible strategy to identify DCM development and slow DCM progression (Figure 5). Although several challenges remain unresolved, epigenetic biomarkers and treatments are under investigation, along with advancing cutting‐edge technologies for basic, translational, and clinical studies.

## Author Contributions

All authors contributed substantially to drafting and revising the manuscript. A.R.H. and N.K. equally contributed to the work as co‐first authors.

## Conflicts of Interest

Y.D. holds patents on RNA biomarkers of cardiovascular disease and is a member of the molecular diagnostics company, Firalis SA. C.J.W. holds patents on epigenetic therapy for cardiomyopathy. Other authors declare no conflicts of interest.

## Peer Review

The peer review history for this article is available at https://www.webofscience.com/api/gateway/wos/peer-review/10.1002/dmrr.70081.

## Data Availability

Data sharing is not applicable to this article as no new data were created or analyzed in this study.
